# Bactericidal, Anti-Biofilm, and Stress-Response Modulatory Effects of *Lacticaseibacillus rhamnosus* ATCC 9595 Cell-Free Supernatant Against *Listeria monocytogenes*

**DOI:** 10.3390/foods14234163

**Published:** 2025-12-04

**Authors:** Isabela Sguilla Rotta, Hugo Felix Perini, Sthefânia Dalva da Cunha Rezende, Yasmin Neves Vieira Sabino, Marcos Vinicius da Silva, Felipe Alves de Almeida, Emiliane Andrade Araujo Naves, Uelinton Manoel Pinto, Alessandra Barbosa Ferreira Machado, Aline Dias Paiva

**Affiliations:** 1 Department of Microbiology, Immunology and Parasitology, Federal University of Triângulo Mineiro, Uberaba 38025-440, Minas Gerais, Brazil; sguillaisabela@gmail.com (I.S.R.); sthenutri@hotmail.com (S.D.d.C.R.); marcos.silva@uftm.edu.br (M.V.d.S.); 2 Department of Education in Science, Mathematics and Technology, Federal University of Triângulo Mineiro, Uberaba 38025-440, Minas Gerais, Brazil; hugo.perini@uftm.edu.br; 3 School of Biological Sciences, Queen’s University Belfast, Belfast BT9 5DL, UK; y.sabino@qub.ac.uk; 4 Department of Microbiology, Institute of Biotechnology Applied to Agriculture, Federal University of Viçosa, Viçosa 36570-900, Minas Gerais, Brazil; felipe.alves@ufv.br; 5 Department of Food Engineering, Federal University of Triângulo Mineiro, Uberaba 38025-180, Minas Gerais, Brazil; emiliane.naves@uftm.edu.br; 6 Laboratory of Food Microbiology, Food Research Center, Department of Food and Experimental Nutrition, Faculty of Pharmaceutical Sciences, University of Sao Paulo, São Paulo 05508-000, Sâo Paulo, Brazil; uelintonpinto@usp.br; 7 Department of Parasitology, Microbiology and Immunology, Federal University of Juiz de Fora, Juiz de Fora 36036-900, Minas Gerais, Brazil; alessandra.machado@ufjf.br

**Keywords:** lactic acid bacteria, foodborne pathogen, biofilm, oxidative stress, virulence gene expression

## Abstract

This study evaluated the antagonistic activity of the cell-free supernatant of *Lacticaseibacillus rhamnosus* ATCC 9595 (*Lcr*-CFS) against *Listeria monocytogenes*, a major foodborne pathogen, that represents a challenge to food safety, due to its remarkable tolerance to environmental stresses and strong biofilm-forming ability. The minimum inhibitory concentration (MIC) and minimum bactericidal concentration (MBC) of *Lcr*-CFS against *L. monocytogenes* were defined as 31.25 and 62.5 mg/mL, respectively. Time-kill assays revealed dose- and time-dependent bactericidal effects. At sub-MICs, *Lcr*-CFS significantly reduced *L. monocytogenes* biofilm formation, disrupted preformed biofilms and decreased cell viability (80.3–96.7%), effects that were confirmed by 3-(4,5-dimethylthiazol-2-yl)-2,5-diphenyltetrazolium bromide (MTT) assay and fluorescence microscopy. Transmission electron microscopy showed *L. monocytogenes* cell wall damage, cytoplasmic leakage, and morphological alterations consistent with bactericidal effects. Additionally, exposure to 1x and 2x MIC of *Lcr*-CFS induced reactive oxygen species (ROS) accumulation, indicating oxidative stress as part of the mechanism by which *Lcr*-CFS exerts its antimicrobial activity. Gene expression analysis revealed upregulation of stress and virulence-associated genes (*sigB*, *prfA*, *degU*, *flaA*, *motA*, *hlyA*, *pclA*, and *actA*) upon exposure to 0.5x MIC suggesting a complex cross-talk network between adaptive mechanisms and environmental stresses. Although *L. monocytogenes* initiates a stress response, it appears unable to counteract the damage induced by *Lcr*-CFS, resulting in cell death. These findings highlight the antimicrobial and anti-biofilm properties of *Lcr*-CFS against *L. monocytogenes*. Given its in vitro efficacy, *Lcr*-CFS emerges as a promising biocontrol agent to improve food safety by mitigating the persistence of *L. monocytogenes* in food processing settings.

## 1. Introduction

*Listeria monocytogenes* is a Gram-positive, rod-shaped, non-spore-forming, and psychrotrophic bacterium that is ubiquitously distributed in the environment [1,2,3,4]. It is recognized as a globally significant foodborne pathogen responsible for both outbreaks and cases of listeriosis, a life-threatening infection acquired through the consumption of contaminated food [5]. Although its incidence is low compared to other foodborne bacterial diseases, listeriosis is associated with high morbidity, with approximately 90% of cases requiring hospitalization, and a mortality rate of 20–30% among high-risk populations, including children, pregnant women, the elderly, and immunocompromised individuals [6,7,8,9].

The ubiquity of *L. monocytogenes* can largely be attributed to the extensive stress adaptations, enabling the bacterium to survive in a variety of harsh environments [10]. Its ability to persist in diverse settings, including food processing and manufacturing facilities, makes *L. monocytogenes* particularly challenging to control and eradicate. As a result, it poses a significant threat to food safety, especially in ready-to-eat products [5,11].

The persistence of *L. monocytogenes* in food processing environments is primarily attributed to its robust biofilm-forming ability, which enhances resistance to sanitizers, heat, desiccation, and acidic stress [12,13]. These biofilms, commonly formed on equipment and surfaces, serve as reservoirs for recurring contamination and significantly hinder sanitation efforts, reinforcing the pathogen’s capacity to endure and disseminate in industrial settings [14,15,16].

The ability of biofilm-associated *L. monocytogenes* to withstand antimicrobial treatments and proliferate in various food matrices underscores the importance of developing effective control strategies to reduce the risk of contamination and safeguard public health [17,18]. In this context, plant-derived compounds, enzymes, bacteriophages, and bacterial metabolites, used alone or in combination with conventional methods, have been evaluated as promising alternative for biofilm control in the food industry [19,20,21,22].

Lactic acid bacteria (LAB) have been extensively investigated for their potential to prevent and control spoilage microorganisms and foodborne pathogens, due to their natural and generally recognized as safe (GRAS) status [23]. LAB are known to produce a diverse arsenal of antimicrobial compounds, such as organic acids, hydrogen peroxide, and antimicrobial peptides [24,25,26].

*Lacticaseibacillus rhamnosus* is a well-characterized LAB widely recognized for its health-promoting effects. Commonly found in the gastrointestinal tract and in fermented dairy products, *Lc. rhamnosus* has the ability to modulate the host immune response, enhance intestinal barrier function, and inhibit the growth of pathogenic microorganisms through the production of antimicrobial compounds such as organic acids, hydrogen peroxide, and bacteriocins [27]. Among its various strains, *Lc. rhamnosus* GG and *Lc. rhamnosus* GR-1 have received particular attention for their probiotic efficacy and safety profile [28,29].

This study aims to assess the unexplored potential of the cell-free supernatant of *Lc. rhamnosus* ATCC 9595 (*Lcr*-CFS) against *L. monocytogenes*. Specifically, we investigated the effects of *Lcr*-CFS on *L. monocytogenes* growth, biofilm formation and disruption, cell wall alterations, oxidative stress induction, and modulation of stress and virulence genes expression.

## 2. Materials and Methods

### 2.1. Bacterial Strains and Culture Conditions

*Lc. rhamnosus* ATCC 9595 was kindly provided by Fiocruz (Fundação Oswaldo Cruz, Rio de Janeiro, Brazil). This strain was routinely grown in Man-Rogosa-Sharpe (MRS) broth (Kasvi, Madrid, Spain), under microaerophilic conditions at 37 °C for 18–24 h. *L. monocytogenes* ATCC 19112, along with strains isolated from food products or food processing environments in Brazil were kindly provided by the *Listeria* Collection (CLIST) of the Laboratory of Bacterial Zoonoses of the Oswaldo Cruz Institute (LABZOO/Fiocruz) (Table 1). *L. monocytogenes* strains were cultivated in Brain Heart Infusion (BHI) broth (Himedia, Mumbai, India), under aerobic conditions at either 28 or 37 °C (depending on the experiment) for 18–24 h. All bacterial strains were preserved at −20 °C in BHI broth containing 20% glycerol.

### 2.2. Lacticaseibacillus rhamnosus ATCC 9595 Cell-Free Supernatant (Lcr-CFS) Obtention, Inhibitory Activity Against Listeria monocytogenes and Degradation by Proteinase K

Stationary-phase cultures of *Lc. rhamnosus* ATCC 9595 (200 mL MRS, 37 °C, 24 h, microaerophilic conditions) [30] were centrifuged (12,500 rpm, 15 min), and the supernatants were filtered through a 0.22 μm pore-size membrane (Kasvi, São José dos Pinhais, Brazil) to obtain the cell-free supernatant (CFS). The CFS was lyophilized (SP Scientific, Warminster, PA, USA) and subsequently rehydrated in phosphate-buffered saline (PBS, pH 7.4) to obtain a stock solution at a final concentration of 500 mg/mL (corresponding to the resuspension of lyophilizate mass). This 500 mg/mL solution derived from the lyophilized CFS was designated as *Lcr*-CFS, stored at −20 °C, and used in all subsequent experiments.

The minimum inhibitory concentration (MIC) of *Lcr*-CFS against *L. monocytogenes* strains was determined by the broth microdilution method according to Clinical and Laboratory Standards Institute guidelines [31]. Aliquots of 50 μL of *Lcr*-CFS and its serial twofold dilutions (prepared from the 500 mg/mL stock solution) were dispensed into the wells of a sterile 96-well microplate containing 50 μL BHI broth (2x concentrated). Subsequently, stationary-phase *L. monocytogenes* cultures (106 CFU/mL; 10 μL) were added. The plates were incubated at 37 °C for 18 h under aerobic conditions [32]. Wells containing uninoculated BHI broth served as negative controls, while medium inoculated solely with *L. monocytogenes* served as growth controls. The MIC was defined as the lowest concentration of *Lcr*-CFS that completely inhibited visible bacterial growth of *L. monocytogenes* after 18 h of incubation at 37 °C.

To determine the minimum bactericidal concentration (MBC), aliquots (5 μL) from wells without visible growth were plated on BHI agar without *Lcr*-CFS and incubated at 37 °C for 48 h. The MBC was defined as the lowest concentration of *Lcr*-CFS yielding no visible colonies, indicating bactericidal activity [33].

To evaluate the proteinaceous nature of the antimicrobial compounds in the *Lcr*-CFS, a proteolytic degradation assay was performed [34], with modifications]. Aliquots of *Lcr*-CFS were incubated with proteinase K (1 mg/mL final concentration; Sigma-Aldrich, St. Louis, MO, USA) at 37 °C for 2 h. The enzyme was inactivated by heating at 95 °C for 10 min. The MIC of the digested *Lcr*-CFS against all *L. monocytogenes* strains was determined as described above. Untreated *Lcr*-CFS (without proteinase K digestion) served as a positive control for antimicrobial activity.

All experimental assays were performed in duplicate.

### 2.3. Time-Kill Assay

Time-kill kinetic studies were performed in BHI broth inoculated with logarithmically grown *L. monocytogenes* 4455 at a final concentration of approximately 10^6^ CFU/mL. *Lcr*-CFS was added at 0.5x, 1x and 2x MIC. Cultures were incubated at 37 °C, and samples were collected at 0, 3, 6, 9, 12, 24, 48 and 72 h to determine viable cell counts in colony forming units per milliliter (CFU/mL) [35]. Time-kill curve was generated by plotting log_10_ CFU/mL versus time (h). Bactericidal activity was defined as ≥99.99% reduction relative to initial bacterial count, while bacteriostatic activity was defined as either maintenance of the initial inoculum or a reduction of <99.99% compared to the initial inoculum [36,37].

### 2.4. Influence of Lcr-CFS on Listeria monocytogenes Biofilm Development and Biofilm Dispersion

Biofilm-forming ability of the *L. monocytogenes* strains selected for this study was previously reported [38], and the bacteria were classified according to the criteria proposed by Stepanóvic et al. [39] into non-, weak, moderate, or strong biofilm producers.

To evaluate the influence of *Lcr*-CFS on biofilm development in 96-well polystyrene microplates, we selected the *L. monocytogenes* 4455, a strong biofilm producer. *L. monocytogenes* 4455 was co-cultured with *Lcr*-CFS at 0.25x and 0.5x MIC in BHI broth at 28 °C, for 72 h, in aerobic conditions. Biofilm biomass was quantified using 0.2% crystal violet staining (30 min), followed by ethanol solubilization and absorbance reading at 540 nm (TP-reader, Thermo Plate, São Paulo, Brazil). Biofilm formation inhibition rates were calculated as follows:

Inhibition rate (%) = [1 − OD540 nm (sample)/OD540 nm (positive control)] × 100.

The effect of *Lcr*-CFS on the bacterial cell viability in mature *L. monocytogenes* biofilms was determined by the 3-(4,5-dimethylthiazol-2-yl)-2,5-diphenyltetrazolium bromide (MTT) assay. After biofilm formation in polystyrene microplates (28 °C, 72 h), wells were washed with PBS, and 100 µL *Lcr*-CFS at different concentrations (0.25 to 2x MIC) were added. The control group received PBS instead of *Lcr*-CFS. Plate was incubated for 24 h at 28 °C [40]. Following incubation, the content of the wells was removed and 10 µL MTT (5 mg/mL) with 90 µL of PBS were added to each well. After 2 h incubation at 37 °C, MTT solution was discarded, and 150 µL of DMSO was immediately added to dissolve the formazan crystals. The plate was then incubated for 15 min at room temperature, and absorbance was measured at 490 nm using a microplate reader (TP-reader, Thermo Plate, São Paulo, Brazil). The experiment was performed in triplicate.

### 2.5. Visual Observation of Biofilm Development on Glass Surfaces in the Presence of Lcr-CFS

Optical microscopy analysis of bacterial biofilm was performed following the method described by Singh et al. [41]. In brief, sterile glass coverslips (1 × 1 cm) were deposited into each well of a 6-well plate, followed by addition of 1.5 mL of BHI broth (2x concentrated), 1.5 mL of *Lcr*-CFS (0.25x or 0.5x MIC), and 300 μL of stationary-phase *L. monocytogenes* 4455 suspension (10^6^ CFU/mL). The plate was incubated (37 °C, 18 h), and planktonic cells were removed by PBS washing (150 μL; 3 times). The remaining biofilms were stained with 0.2% crystal violet for 30 min [42,43]. Biofilms were examined under light microscope (Nikon Instruments, New York, NY, USA) at 400× magnification for overall biofilm analysis and at 1000× for visualization of individual cell attachment. All experimental assays were performed in duplicate.

### 2.6. Viability of L. monocytogenes Cells in Mature Biofilms After Lcr-CFS Treatment

Mature biofilms were developed on sterile glass coverslips placed in 6-well plates, as described above, in the absence of *Lcr*-CFS. The mature biofilms were submerged with *Lcr*-CFS (0.25x to 2x MIC) for 24 h, and stained with propidium iodide (PI) (10 μg/mL, 10 min). PI selectively stains dead cells or those with compromised membranes, emitting red fluorescence [44]. Biofilms non-exposed to *Lcr*-CFS were used as a control. Fluorescent images were acquired using an inverted fluorescence microscope EVOS (Invitrogen, Waltham, MA, USA) equipped with a 40x phase-contrast objective and a ICX285AL CCD camera (Sony, Hod Hasharon, Israel) [40]. All tests were performed in duplicate.

### 2.7. Reactive Oxygen Species Production Induced by Lcr-CFS Exposure

Aliquots of 30 μL of *L. monocytogenes* 4455 (10^6^ CFU/mL) were exposed to 300 μL of *Lcr*-CFS (0.25x to 2x MIC) for 3 h at 37 °C. After incubation, cells were harvested by centrifugation (3500 rpm, 10 min), the supernatant was discarded, and the pellet was suspended in 300 µL of PBS. Cell suspensions were stained with 0.1 μM 2′,7′-dichlorodihydrofluorescein diacetate (DCFH-DA; Sigma-Aldrich, USA) and incubated at 37 °C for 20 min. Following staining, cells were centrifuged (3500 rpm, 10 min) and washed twice using PBS to remove the extracellular dye. Aliquots of each treatment (100 μL) were transferred to polystyrene microplates and fluorescence intensity was measured using a fluorescence microplate reader EnSpire^®^ (Perkin Elmer, Rodgau, Germany) with excitation at 485 nm and emission at 535 nm [45]. All experimental assays were performed in triplicate.

### 2.8. Effect of Lcr-CFS Treatment on Listeria monocytogenes Cell Structure

*L. monocytogenes* 4455 (10^6^ CFU/mL) was incubated with *Lcr*-CFS at 0.5x and 1x MIC, at 37 °C for 24 h, to assess potential damages to the bacterial cell wall. Following incubation, cells were centrifuged (12,000 rpm, 10 min), washed three times with sodium phosphate solution (0.1 M, pH 7.2; 1 mL), and fixed in 2.5% glutaraldehyde and 4% paraformaldehyde in PBS. Post-fixation was performed with 0.1% osmium tetroxide and 0.1% ruthenium red for 3 h. Cells were dehydrated through graded ethanol series (50, 70, 90, 95, and 100% for 10 min each), followed by three washes in absolute ethanol and three washes in pure acetone for 20 min. EMbed 812 resin (EMS-14120) was added in increasing concentrations in acetone (2:1 overnight; 1:1 for 4 h; and pure resin for 48 h at 60 °C) according to the method described by Liu et al. [46]. Ultrathin sections were placed on copper grids and observed using a Zeiss EM 900 transmission electron microscope (Zeiss, Oberkochen, Germany; Electronic Microscopy Laboratory—UFTM) at 5000× magnification.

### 2.9. Influence of Lcr-CFS on L. monocytogenes Gene Expression

The effect of *Lcr*-CFS on the expression of the genes *prfA*, *sigB*, *degU*, *flaA*, *motA*, *hlyA*, *pclA* and *actA* was evaluated by real-time quantitative reverse transcription polymerase chain reaction (RT-qPCR). *L. monocytogenes* 4455 cultures (10^6^ CFU/mL), and 0.5x MIC of *Lcr*-CFS were mixed and incubated at 28 °C for 24 h. Following incubation, cultures were centrifuged (13,500 rpm, 5 min.) and washed with PBS (1 M, pH 7.0). Total RNA was extracted using Trizol reagent (Sigma-Aldrich, St. Louis, MO, USA), and complementary DNA (cDNA) was synthesized with the High-Capacity cDNA Reverse Transcription Kit (Thermo Fisher, Waltham, MA, USA). Gene expression was quantified by RT-qPCR and performed with PowerTrack^TM^ SYBR Green Master Mix for qPCR (Thermo Fisher, USA), following the manufacturer’s instructions. Primers used in this study were previously reported (Table 2) and the 16S rRNA gene was used as a housekeeping control.

Each reaction contained of the following components: 5 µL of PowerTrack SYBR Green Master Mix, 0.5 µL of forward primer, 0.5 µL of reverse primer, 0.25 µL of yellow dye, 1 µL of cDNA (20 ng), and 3.25 µL of nuclease-free water, resulting in a final reaction volume of 10 µL. Amplification was carried out using the StepOnePlus^TM^ Real-Time PCR System (Thermo Fisher, USA). Cycling conditions were as follows: an initial denaturation step at 95 °C for 2 min, followed by 40 cycles of 95 °C for 15 s and 60 °C for 60 s. The specificity of the amplification was confirmed by melting curve analysis. Relative gene expression levels were calculated using the comparative CT method (2^−ΔΔCt^) [50]. All experiments were performed in biological duplicates and experimental triplicates.

### 2.10. Statistical Analysis

Statistical analyses were performed using GraphPad Prism 8.0 software (GraphPad Software Inc., La Jolla, CA, USA). All data were subjected to the F test and the Shapiro–Wilk test to assess variance and normality, respectively. Comparisons between groups were conducted using one-way ANOVA, followed by Dunnett’s test to evaluate the effect of *Lcr*-CFS on biofilm formation. Differences were considered statistically significant at *p* < 0.05. For the analysis of ROS production and relative gene expression, ANOVA was also employed. Genes were considered to be upregulated (2^−ΔΔCt^ > 1) or downregulated (2^−ΔΔCt^ < 1) in the presence of *Lcr*-CFS when statistically significant differences (*p* < 0.05) were observed in Ct values between the control and the treated groups.

## 3. Results

### 3.1. Bactericidal Activity of Lcr-CFS Against Listeria monocytogenes

The cell-free supernatant obtained from stationary-phase cultures of *Lc. rhamnosus* ATCC 9595 exhibited an initial pH of 4.5. The *Lcr*-CFS was subsequently lyophilized and suspended in PBS (pH 7.4) prior to use. The inhibitory activity of *Lcr*-CFS was assessed by determining its MIC against twenty-one *L. monocytogenes* strains. The growth of all bacterial strains was completely inhibited by *Lcr*-CFS, and a concentration of 31.25 mg/mL was determined as the MIC. The MBC was 62.5 mg/mL, corresponding to twice the MIC (2x MIC), suggesting a potential bactericidal effect of *Lcr*-CFS.

**Figure 1:**
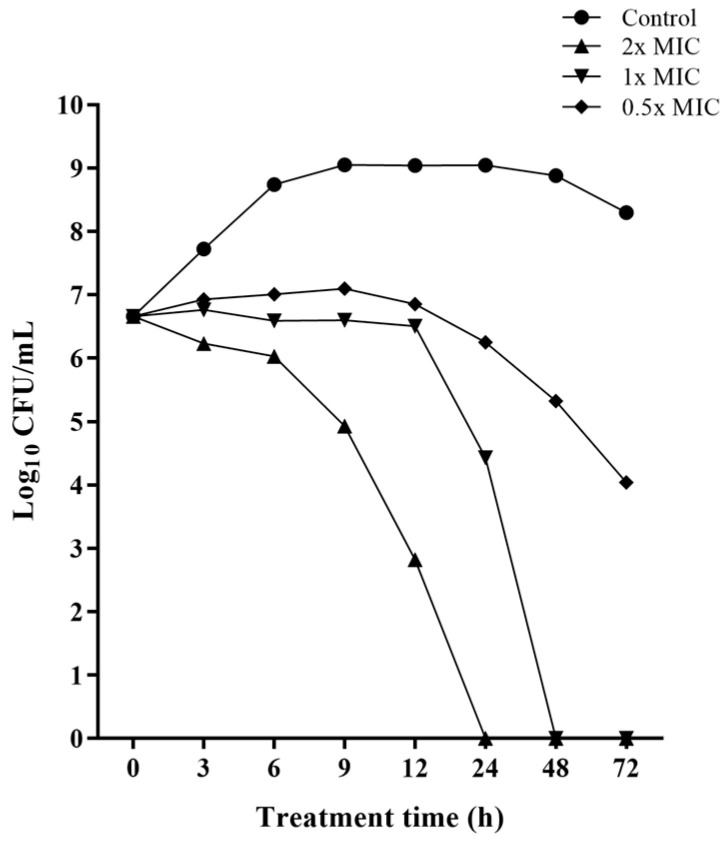
Time-kill curves demonstrating the bactericidal activity of *Lacticaseibacillus rhamnosus* cell-free supernatant (*Lcr*-CFS) against *Listeria monocytogenes* 4455. *Lcr*-CFS was added at time point zero (0.5x, 1x, and 2x MIC), and UFC/mL was determined for up to 72 h. The control curve represents bacterial growth in the absence of *Lcr*-CFS.

The bactericidal activity of *Lcr*-CFS against all *L. monocytogenes* strains was further confirmed by time-kill experiments, showing dose- and time-dependent kinetics. Co-incubation of *L. monocytogenes* with *Lcr*-CFS at the MBC (62.5 mg/mL) resulted in an approximately 4-log reduction in the initial bacterial population after 12 h. At the MIC (31.25 mg/mL), *L. monocytogenes* growth was inhibited for 12 h, with effective cell death observed at 24 h of incubation (approximately 3-log reduction), and absence of colonies after 48 h of exposure. At 0.5x MIC (15.6 mg/mL), an approximately 3-log reduction in *L. monocytogenes* population was observed after 72 h of exposure to *Lcr*-CFS (Figure 1).

*Lcr*-CFS markedly inhibited *L. monocytogenes* growth, resulting in an optical density (OD_600_) of 0.131. In contrast, after treatment with proteinase K, *Lcr*-CFS lost its inhibitory activity, and the growth of *L. monocytogenes* was comparable to the untreated control (OD_600_ of 0.435 vs. 0.495, respectively). This result strongly suggests that the bioactive compound (s) synthesized by *Lc. rhamnosus* is proteinaceous in nature and sensitive to degradation by proteinase K.

### 3.2. Anti-Biofilm Effects of Lcr-CFS

As recently reported by our research group [38], *L. monocytogenes* 4455 (serotype 1/2a), isolated from a frozen chicken carcass in 2017, is a strong biofilm producer. Thus, strain 4455 was selected as representative of the other *L. monocytogenes* strains for the experiments that aimed to analyze the anti-biofilm effects of *Lcr*-CFS.

At 0.25x and 0.5x MIC, *Lcr*-CFS significantly reduced biofilm formation by *L. monocytogenes* 4455 compared to the untreated group (biofilm formed in the absence of *Lcr*-CFS) (*p* < 0.05). At concentrations equal or above the MIC, no bacterial growth was observed (Table 3). *Lcr*-CFS also disrupted pre-formed *L. monocytogenes* biofilms, reducing cell viability as indicated by MTT conversion relative to the untreated control biofilm (*p* < 0.05). It is important to highlight that the effect of *Lcr*-CFS on disrupting mature biofilms did not significantly differ among the tested concentrations, with reductions in cell viability ranging from 80.3 to 96.7% at 0.25x and 2x MIC, respectively (*p* > 0.05) (Table 3).

**Figure 2:**
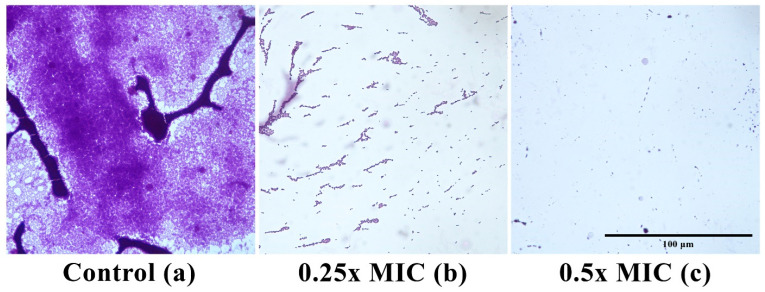
Optical microscope images of 72 h *Listeria monocytogenes* 4455 biofilm grown on glass surfaces stained with crystal violet. (**a**) biofilm formed in the absence and presence of *Lacticaseibacillus rhamnosus* ATCC 9595 (*Lcr*-CFS) at 0.25x MIC (**b**) and 0.5x MIC (**c**). Magnification 400×. Scale bar is 100 µm.

Visualization by light microscopy revealed well-formed and compact biofilms formed by *L. monocytogenes* 4455 on glass surfaces after 72 h of incubation at 28 °C (Figure 2a). In contrast, co-culture with *Lcr*-CFS at 0.25x and 0.5x MIC inhibited the development of *L. monocytogenes* biofilms, with only scattered cells being visualized (Figure 2b,c).

**Figure 3:**
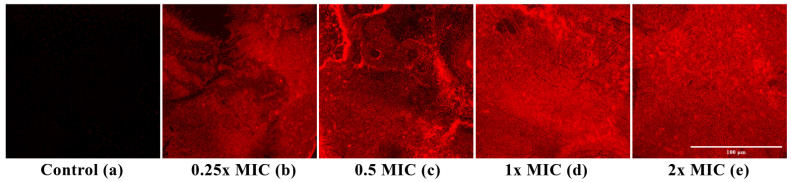
Fluorescent microscope images of *Listeria monocytogenes* 4455 biofilms formed on glass surfaces after 72 h. (**a**) control (non-treated, non-fluorescent cells); (**b**–**e**) mature biofilms treated with *Lcr*-CFS (0.25x–2x MIC). Biofilms were stained with propidium iodide (PI), and red color indicates dead bacterial cells on glass surfaces. Magnification 40×. Scale bar is 100 µm.

The ability of *Lcr*-CFS to disrupt mature *L. monocytogenes* biofilms formed on glass surfaces for 72 h was analyzed by fluorescence microscopy using PI, which selectively penetrates cells with compromised membranes and serves as a marker of cell death. Treatment with *Lcr*-CFS at 0.25x, 0.5x, 1x, and 2x MIC (Figure 3b–e) resulted in a marked increase in PI fluorescence intensity compared to the untreated control (Figure 3a), indicating bacterial cell death within the biofilm.

### 3.3. Induction of Reactive Oxygen Species (ROS) by Lcr-CFS

**Figure 4:**
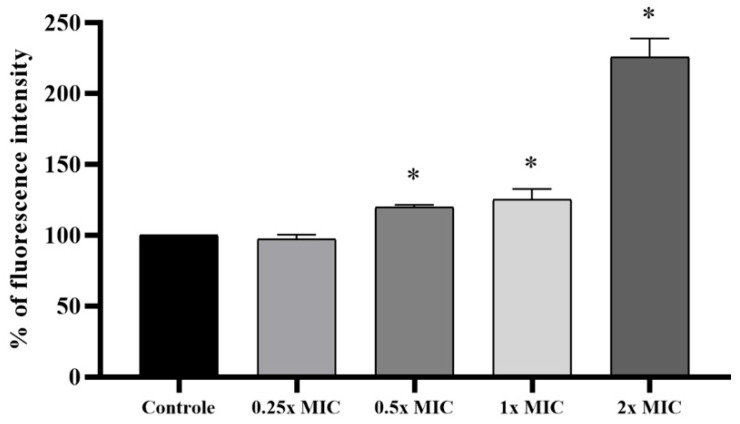
Induction of reactive oxygen species (ROS) in *Listeria monocytogenes* 4455 treated with *Lacticaseibacillus rhamnosus* cell-free supernatant (*Lcr*-CFS) at concentrations of 0.25x to 2x MIC. Means marked with an asterisk (*) indicate significant differences compared to the control group, based on Dunnett’s test (*p* < 0.05).

Treatment with *Lcr*-CFS at 1x and 2x MIC significantly enhanced ROS production in *L. monocytogenes* 4455 compared to the control condition (Figure 4). Notably, exposure to 2x MIC resulted in markedly higher fluorescence intensity (2.26-times fold), indicating substantial ROS generation and induction of oxidative stress. The accumulation of ROS induced by *Lcr*-CFS can lead to the damage of *L. monocytogenes* cells by either direct reaction with biomolecules such as proteins, lipids, and nucleic acids, or to indirect regulation of signaling pathways.

### 3.4. Effect of Lcr-CFS Treatment on Listeria monocytogenes Cell Structure

**Figure 5:**
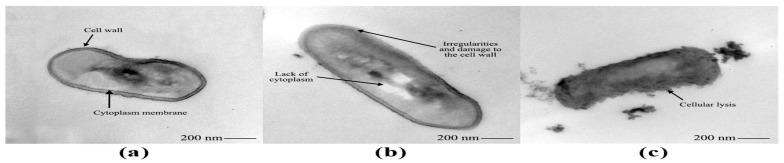
Transmission electron microscope images of *Listeria monocytogenes* 4455 cells before (**a**) and after exposure to *Lacticaseibacillus rhamnosus* ATCC 9595 cell-free supernatant (*Lcr*-CFS) at 0.5x MIC (**b**) and 1x MIC (**c**).

Under transmission electron microscopy, untreated *L. monocytogenes* 4455 cells exhibited normal rod-shaped morphology, a typical and continuous cell wall, an intact and smooth cytoplasmic membrane, as well as a cytoplasm evenly distributed along the cell (Figure 5a). After exposure to *Lcr*-CFS at 1x MIC for 24 h, bacterial cell lysis and leakage of intracellular material were evidenced (Figure 5c). Treatment with 0.5x MIC of *Lcr*-CFS caused irregularities, damage and deformation on *L. monocytogenes* cell wall, in addition to partial loss of cytoplasm (Figure 5b).

### 3.5. Changes in Gene Expression Induced by Lcr-CFS

**Figure 6:**
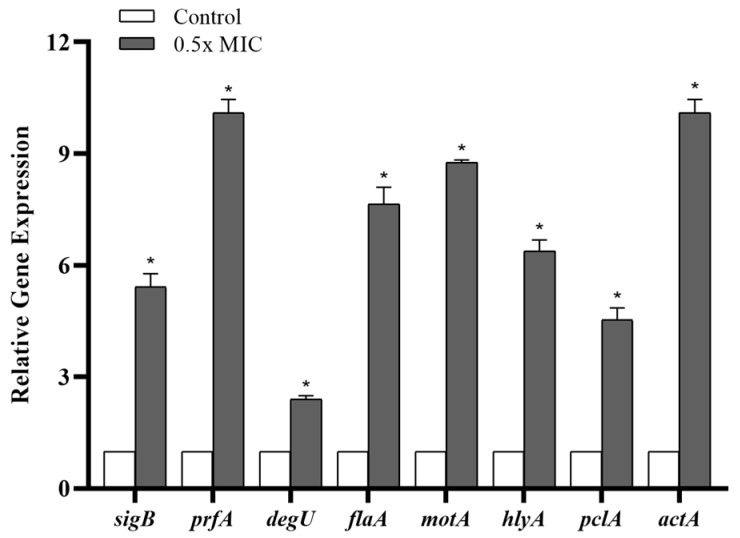
Relative gene expression in *Listeria monocytogenes* 4455 after 24 h of exposure to 0.5x MIC of *Lacticaseibacillus rhamnosus* ATCC 9595 cell-free supernatant (*Lcr*-CFS). Means marked with an asterisk (*) indicate significant differences compared to the control group (non-exposed to *Lcr*-CFS), based on ANOVA with Tukey’s post-test (*p* < 0.05).

Taking into account the bactericidal activity of the *Lcr*-CFS at MIC-level concentrations or above, the relative expression of genes associated with response regulators (*prfA*, *sigB*, and *degU*), flagella (*flaA*, *motA*), and virulence factors (*hlyA*, *pclA*, and *actA*) was evaluated in *L. monocytogenes* 4455 upon exposure to 0.5x MIC of *Lcr*-CFS for 24 h. Compared to untreated bacterial cells, the expression of all evaluated genes was significantly upregulated in the presence of *Lcr*-CFS (*p* < 0.05). Notably, the transcription level of *prfA*, the main regulator of virulence factors in *L. monocytogenes*, and *sigB*, the primary stress response regulator, were significantly elevated (*p* < 0.05) following treatment with *Lcr*-CFS (Figure 6).

## 4. Discussion

Lactic acid bacteria (LAB) are recognized as effective biological control agents against the growth of undesirable microorganisms. Their protective effects primarily result from the production of antimicrobial metabolites, including organic acids, hydrogen sulfide, hydrogen peroxide, exopolysaccharides, biosurfactants, and antimicrobial peptides. Additionally, LAB can compete for nutrients [18,51] and antagonize the formation of biofilms by pathogenic microorganisms through mechanisms such as competition, exclusion, or displacement [52,53].

Several bioactive compounds produced by LAB, including antimicrobial metabolites, can be directly recovered from the culture supernatant without extensive purification processes. This highlights the potential of cell-free supernatants (CFS) as a convenient and efficient source of bioactive metabolites for diverse applications. Antimicrobial activity of CFS from different bacteria has been reported against pathogenic microorganisms [38,54,55,56,57,58,59,60,61,62].

In this study, we demonstrated the anti-*Listeria* activity of the CFS obtained from *Lc. rhamnosus* ATCC 9595. The supernatant obtained from stationary-phase cultures had an initial pH of 4.5; however, since the *Lcr*-CFS was lyophilized and suspended in PBS (pH 7.4), the observed anti-*Listeria* activity likely did not result from organic acid production. Furthermore, after treatment with proteinase K, CFS lost its anti-listerial activity, demonstrating the proteinaceous nature of the compound produced by *Lc. rhamnosus*.

Many LAB are known to produce ribosomally synthesized peptides, named bacteriocins, which exhibit bacteriostatic or bactericidal activity. Among these, pediocin-like class IIa bacteriocins are particularly effective against *Listeria* [63]. *Lc. rhamnosus* strains have been reported to produce bacteriocins, including compounds with anti-listerial activity [64,65,66]. The hypothesis that the anti-*Listeria* compound produced by *Lc. rhamnosus* ATCC 9595 is a bacteriocin or a bacteriocin-like substance is supported by the recent sequencing of its genome, which revealed genes encoding bacteriocin immunity proteins and accessory proteins involved in bacteriocin secretion [67].

The bactericidal activity of *Lcr*-CFS, observed in MBC and time-kill assays, was confirmed by transmission electron microscopy. *L. monocytogenes* cells exposed to 1x MIC of *Lcr*-CFS exhibited structural alterations, including damage to the bacterial cell envelope, membrane destabilization, and leakage of intracellular compounds. In contrast, exposure to 0.5x MIC resulted in structural changes and damage to the bacterial cell wall, indicating that, even at concentrations lower than the MIC, *Lcr*-CFS may disrupt vital cellular functions and ultimately cause bacterial cell death.

Antibacterial peptides produced by LAB commonly act by forming pores in the target bacterial cell membrane, which leads to membrane permeability increase, depolarization and leakage of intracellular compounds [68,69,70,71]. It has also been demonstrated that LAB and CFSs derived from LAB can elevate reactive ROS levels in target cells [72,73,74]. Recently, Wang and Zeng [45] demonstrated that the CFS from *Lactobacillus pentosus* L-36 exhibits antibacterial effects by damaging cell membranes and increasing ROS levels in *Staphylococcus aureus*.

Exposure to *Lcr*-CFS increased ROS production in *L. monocytogenes*, indicating that its lethal action derives, at least in part, from ROS-mediated oxidative stress. ROS have been associated with the lethal action of different antibiotics, regardless of their specific targets [75,76]. Large quantities of ROS can overwhelm the endogenous antioxidant defenses of pathogens, directly damaging DNA and other cellular components, leading to cell death [77,78]. Moreover, cell death can occur due to post-stress ROS-mediated toxicity if the primary damage induced by the stressor was insufficient to kill bacteria due to repair. Supporting this hypothesis, evidence from *Escherichia coli* was provided by Hong et al. [79], whose findings showed that even after removal of stressor, ROS continued to accumulate, leading to ongoing cell death, indicating that once a ROS level exceeds a critical threshold, the death process becomes self-driven.

Beyond its bactericidal activity, *Lcr*-CFS exhibited potent anti-biofilm effects against *L. monocytogenes*, even at sub-inhibitory concentrations (sub-MIC), inhibiting biofilm formation/development and affecting preformed biofilms. A complete inhibition of *L. monocytogenes* biofilm formation was achieved in the presence of 0.5x MIC *Lcr*-CFS, both in polystyrene microplates and on glass surfaces. This effect can be due to the ability of *Lcr*-CFS in reducing bacterial adhesion during the initial stages of biofilm development or to prevent the transition to the biofilm phenotype. The anti-biofilm activity of LAB has been reported in numerous studies [3,38,42,80,81,82,83,84,85,86].

Comparable anti-biofilm effects were reported by Singh et al. [53], who showed that CFS from various *Lactobacillus* strains effectively inhibited *L. monocytogenes* biofilm development. In co-incubation assays, the most notable reduction in *L. monocytogenes* biofilm formation was obtained using *Lc. rhamnosus* GG (63.01%) and *Lc. rhamnosus* GM18 (62.72%) supernatants. A significant reduction (more than 65.0%) in the development of biofilms by *L. monocytogenes* was also reported in the presence of CFS obtained from *Lactobacillus acidophilus* La14 150B, *Lactiplantibacillus plantarum* B411, and *Lacticaseibacillus rhamnosus* ATCC 53103 [87].

Recently, our research group demonstrated the ability of CFS of *Weissella paramesenteroides* UFTM 2.6.1 in inhibiting biofilm formation by *L. monocytogenes*: at concentrations equal to or above the MIC a complete inhibition (100.0%) of biofilm formation was observed, while a sub-MIC (0.5x MIC) reduced biofilm biomass by 59.8% to 89.5%, depending on the *L. monocytogenes* strain [38].

Melian et al. [88] reported that treatment of *L. monocytogenes* biofilms with lactocin AL705 (800 AU/mL), produced by *Latilactobacillus curvatus* CRL1579, for 30 min resulted in a 5-log reduction in viable cell counts and a decrease in biofilm biomass, with microscopy confirming a predominance of dead or damaged cells. Yan et al. [89] demonstrated that treatment with nisin, a bacteriocin produced by a group of Gram-positive bacteria, significantly reduced preformed *L. monocytogenes* biofilms. After 2 h of exposure, bacterial concentration was decreased by 23.94–35.67% compared to the untreated control.

Our findings demonstrated that *Lcr*-CFS significantly reduced the viability of *L. monocytogenes* cells within the biofilms formed in polystyrene plates, with concentrations equal to or higher than 0.25x MIC leading to a reduction of more than 80% in cell viability. Additionally, when preformed *L. monocytogenes* biofilms on glass surfaces were treated with *Lcr*-CFS, fluorescence microscopy revealed extensive cell death within the biofilm matrix, as indicated by the predominance of red-stained (nonviable) cells compared to the untreated control.

The ability of *L. monocytogenes* to survive in highly variable and often hostile environments relies on its finely tuned capacity to rapidly reprogram gene expression, a process largely mediated by alternative sigma factors [18,90,91]. Among these, the stress-responsive sigma factor B (SigB; σB) is crucial for enabling *L. monocytogenes* to adapt its transcriptional profile. Notably, the transcriptional program regulated by SigB is intricately linked with PrfA, the primary regulator of virulence genes, forming a complex network that allows *L. monocytogenes* to coordinate stress responses and pathogenicity simultaneously [92]. This coordinated regulation of stress adaptation and virulence maintains the bacterium’s ability to survive and persist in the natural environment, the food chain and within the host [10,93,94,95,96].

Exposure to *Lcr*-CFS 0.5x MIC significantly upregulated *sigB* and *prfA* genes in *L. monocytogenes*, reflecting a coordinated activation of stress response and virulence-associated pathways. Importantly, this transcriptional response likely represents an adaptative mechanism to cope with antimicrobial stress rather than a true increase in virulence or pathogenic potential. The observed cross-talk between *sigB* and *prfA* underscores the plasticity of *L. monocytogenes* regulatory networks, allowing the bacterium to modulate stress adaptation and virulence genes simultaneously in response to environmental challenges [10,95].

Upregulation of *prfA* concomitant with reduced biofilm formation after curcumin-mediated photodynamic inactivation of *L. monocytogenes* were also reported by Huang et al. [97], who suggested that the treatment increased the stress responses of the *L. monocytogenes* cells to resist external harsh environment. A comparable increase in *sigB* and *prfA* expressions was also observed in our recent study using the CFS of *W. paramesenteroides* UFTM 2.6.1, suggesting a conserved stress-related response in *L. monocytogenes* induced by bioactive compounds produced by LAB [38].

Additionally, the upregulation of other genes, including response regulators (*degU*), flagellar-associated genes (*flaA* and *motA*), and virulence factors (*hlyA*, *pclA*, and *actA*), following exposure to the *Lcr*-CFS likely reflects a bacterial survival strategy in response to antimicrobial stress, rather than an actual increase in virulence or biofilm-forming capacity, highlighting the complex and variable impact that antimicrobial agents can exert on bacterial regulatory networks. In line with our observations, Liu et al. [32] demonstrated the upregulation of genes associated with motility (*degU*, *flaA*, *motB*), stress response (*sigB*), and virulence (*prfA*) after treatment with natural antimicrobials, such as cinnamaldehyde, eugenol, resveratrol, and thymoquinone at sub-MICs.

Although the experiments reported in this study were conducted exclusively using in vitro models, these conditions were strategically selected to elucidate the fundamental effects of *Lcr*-CFS against *L. monocytogenes*, and findings reported provide essential preliminary evidence supporting the biocontrol potential of *Lcr*-CFS. The lack of experiments in more realistic food-relevant environments represents an important limitation and should be considered for validating *Lcr*-CFS applicability in food safety strategies.

## 5. Conclusions

Bioactive compounds secreted by *Lc. rhamnosus* ATCC 9595 (*Lcr*-CFS) exhibits potent bactericidal and anti-biofilm effects against *L. monocytogenes*. As the supernatant contains a complex mixture of bioactive compounds, the anti-*Listeria* effect of *Lcr*-CFS is likely the result of synergistic action of multiple mechanisms rather than a single dominant pathway. The bactericidal potential appears to be associated with bacterial cell wall disruption and intracellular leakage, besides induction of ROS, whereas its anti-biofilm activity likely results from reduced adhesion of planktonic cells to plastic and glass surfaces, and disruption of mature biofilms, leading to loss of *L. monocytogenes* cells viability. Although at concentrations lower than the MIC (0.5x), *Lcr*-CFS induced upregulation of genes associated with stress response and virulence in *L. monocytogenes*, these transcriptional changes did not translate into measurable phenotypic alterations, being insufficient to overcome *Lcr*-CFS bactericidal and anti-biofilm effects. Given these properties, *Lc. rhamnosus* represents a promising biocontrol agent in food industry settings to mitigate *L. monocytogenes* contamination and persistence.

## Figures and Tables

**Table 1:** *Listeria monocytogenes* strains: code, serotypes, year of the isolation, sources, and specimen types.

Code	Serotype	Year of Isolation	Source	Specimen Type
706	4b	2011	Food	Yakisoba
1018	4b	2011	Food	Mini pizza
1071	4b	2009	Food	Powdered milk
3492	4b	2013	Environment	Meat tenderizer
3803	4bb	2014	Food	Organic vegetables (Beetroot)
3833	1/2b	2014	Environment	Industrial floor
3837	4b	2014	Environment	Drain processing room
3839	1/2b	2014	Environment	Drain processing room
3992	1/2b	2015	Food	Chicken liver pate
4001	1/2c	2015	Food	Beef
4107	1/2a	2015	Food	Temaki Philadelphia restaurant C
4251	1/2b	2016	Food	Packaged chicken thigh
4313	1/2b	2016	Environment	Dough preparation drain swab
4330	1/2b	2016	Environment	Argentine slicer belt swab
4449	1/2a	2017	Environment	Boards and knives swab
4455	1/2a	2017	Food	Frozen chicken carcass
4484	1/2c	2017	Food	Frozen chicken meat cuts
4506	4b	2017	Environment	Table with mats

**Table 2:** Primer pairs used in real-time quantitative reverse transcription polymerase chain reactions (RT-qPCR).

Gene	Primer	Sequences (5′-3′)	Reference
*16S rRNA*	F R	CCGTCAAGGGACAAGCA GGGAGGCAGCAGTAGGGA	[47]
*sigB*	F R	TGGATTGCCGCTTACCAAGAA TCGGGCGATGGACTCTACTA	[48]
*prfA*	F R	AGAAACATCGGTTGGCTATT TTGACCGCAAATAGAGCC	[47]
*degU*	F R	ACGCATAGAGAGTGCGAGGTATT CCCAATTCCGCGGTTACTT	[48]
*flaA*	F R	GGCTGCTGAAATGTCCGAAA TGCGGTGTTTGGTTTGCTTG	[48]
*motA*	F R	TTTTACGGGATGTTTTGGAA TCGCTAAGTTTGTCTGGGTT	[49]
*hlyA*	F R	TGACGAAATGGCTTACAGT TTTTCCCTTCACTGATTGC	[47]
*pclA*	F R	TACTCCCAGAACTGACACGA CTCGGACCATTGTAGTCATCT	[48]
*actA*	F R	CCTGTAAAGACCGCACCA GCTGATTCGCTTTCCTCTAC	[48]

**Table 3:** Effect of different concentrations of cell-free supernatant of *Lacticaseibacillus rhamnosus* ATCC 9595 (*Lcr*-CFS) on biofilm formation and on disruption of mature biofilm of *Listeria monocytogenes* 4455 on polystyrene surfaces measured by crystal violet and MTT staining, respectively.

*L. monocytogenes* 4455	Biofilm Formation		Mature Biofilm	
	OD_540_	Reduction (%)	OD_490_	Reduction (%)
Control	0.85 ± 0.11		0.61 ± 0.02	
0.25x MIC	0.02 ± 0.02	97.6 *	0.12 ± 0.02	80.3 *
0.5x MIC	0.00 ± 0.01	100.0 *	0.07 ± 0.08	88.5 *
1x MIC	-	-	0.05 ± 0.04	91.8 *
2x MIC	-	-	0.02 ± 0.03	96.7 *

Results are expressed as mean ± standard deviations of three independent experiments. Means marked with an asterisk (*) indicate significant differences compared to the control group, based on Dunnett’s test (*p* < 0.05). (-) absence of bacterial growth.

## Data Availability

The original contributions presented in this study are included in the article. Further inquiries can be directed to the corresponding author.

## Abbreviations

The following abbreviations are used in this manuscript:

*Lcr*-CFS	Cell-Free Supernatant of *Lacticaseibacillus rhamnosus* ATCC 9595
CFS	Cell-Free Supernatant
LAB	Lactic acid bacteria
GRAS	Generally Recognized As Safe
FIOCRUZ	Fundação Oswaldo Cruz
MRS	Man-Rogosa-Sharpe
CLIST	*Listeria* Collection
LABZOO	Laboratory of Bacterial Zoonoses of the Oswaldo Cruz Institute
BHI	Brain Heart Infusion
PBS	Phosphate-Buffered Saline
MIC	Minimum Inhibitory Concentration
CLSI	Clinical and Laboratory Standards Institute
MBC	Minimum Bactericidal Concentration
CFU/mL	Viable cell counts in colony forming units per milliliter
MTT	3-(4,5-dimethylthiazol-2-yl)-2,5-diphenyltetrazolium bromide
PI	Propidium Iodide
DCFH-DA	2′,7′-dichlorodihydrofluorescein diacetate
RT-qPCR	Real-Time Quantitative Reverse Transcription Polymerase Chain Reaction
RNA	Ribonucleic Acid
DNA	Deoxyribonucleic Acid
cDNA	Complementary DNA
ROS	Reactive oxygen species
OD	Optical Density
